# Perception of the Movement Control Order during the COVID-19 Pandemic: A Qualitative Study in Malaysia

**DOI:** 10.3390/ijerph18168778

**Published:** 2021-08-20

**Authors:** Yea Lu Tay, Zalilah Abdullah, Kalvina Chelladorai, Lee Lan Low, Seng Fah Tong

**Affiliations:** 1 Institute for Health Systems Research, National Institutes of Health, Ministry of Health Malaysia, Shah Alam 40170, Malaysia; zalilah.a@moh.gov.my (Z.A.); drkalvina@moh.gov.my (K.C.); low.ll@moh.gov.my (L.L.L.); 2 Department of Family Medicine, Faculty of Medicine, Universiti Kebangsaan Malaysia, Kuala Lumpur 56000, Malaysia; tsf@ppukm.ukm.edu.my

**Keywords:** lockdown, Movement Control Order, pandemic, COVID-19, communications, public health measures

## Abstract

Malaysia implemented its first Movement Control Order (MCO) during the early phase of the COVID-19 pandemic to slow the transmission of the virus. This study aimed to explore the public perception of the MCO implementation and people’s experiences during this period. The study employed qualitative explorative in-depth interviews conducted with 23 Malaysian adults from various demographic backgrounds. Thematic analysis was performed using NVivo 12. Three main themes were identified: a period of information surge, heterogeneous emotional response, and attempts to adapt. During the MCO, the participants obtained information from multiple platforms. They suggested the need for clear and repeated instructions to avoid confusion and misinformation. They also acknowledged the importance of the MCO in breaking the chain of transmission and safeguarding high-risk groups; however, they also expressed that stricter enforcement from the authorities was warranted. The changes in the participants’ work–life routines, lack of physical interaction, and uncertainty about their health and the economy due to the MCO negatively impacted their psychological states. Despite these challenges, the participants attempted to adapt to life under the MCO in different ways. The findings imply that during a crisis, the public tends to seek clear and reliable information, experience emotional turmoil, and adapt to changes. The MCO implementation can be improved through an effective communication strategy and efforts to battle misinformation.

## 1. Introduction

Coronavirus disease 2019 (COVID-19) was first detected in the city of Wuhan, China, at the end of December 2019, and presented as an outbreak of pneumonia with unknown etiology [1]. Following the worldwide spread of the disease, the World Health Organization (WHO) declared the outbreak a global pandemic on 11 March 2020 [2]. As of 14 October 2020, the disease had spread to 217 countries and territories globally, affecting more than 38 million individuals, with over a million deaths recorded [3].

The lack of specific treatments or vaccines during the early phase of the pandemic prompted many countries to implement various public health interventions, ranging from physical distancing recommendations to stricter lockdown measures in the attempt to flatten the COVID-19 infection curve, which was essential to prevent overwhelmed health systems [4,5,6]. Nevertheless, different lockdown rules were applied by every country [7,8]. In general, lockdown can be described as a series of interventions aiming to restrict movement and social interactions, thus suppressing the transmission of COVID-19 [9].

Some countries imposed geographic containment, also known as cordon sanitaire, by limiting individuals’ movement into and out of disease hotspots. Geographic containment aimed to prevent the spread of the disease from the hotspots, such as Wuhan in January 2020 or Northern Italy in March 2020, to other parts of the nation or region. This measure was also expected to curb the import of infections from high-risk areas or countries [10,11,12]. In addition, many countries implemented stringent physical distancing measures through home confinement, also known as shelter-in-place intervention, aiming to slow down the rapid transmission of the virus [13,14]. Citizens were required to stay at home for a specified period unless they worked in essential services or needed to replenish food supplies or seek medical services. All non-essential services, such as businesses, schools, universities, entertainment premises, religious places, and sports facilities, were closed, and public gatherings were restricted [12,14].

### Movement Control Order in Malaysia

In Malaysia, the exponential spike of COVID-19 cases following a community outbreak related to a religious mass gathering in Kuala Lumpur prompted the government to take a major step by implementing its first-ever set of nationwide lockdown measures, known as the Movement Control Order (MCO), on 18 March 2020 [15]. The order was implemented pursuant to the Prevention and Control of Infectious Diseases Act 1988 (Act 342) and the Police Act 1967 [15,16]. During the MCO period, the Malaysians were urged to stay at home, and mass movements and gatherings of all kinds were completely prohibited across the country. All government and private education institutions and premises were ordered to close, except for those involved in essential services, such as utilities, healthcare services, emergency services, food and daily necessities, transportation, and logistics, as well as banking and finance. In addition, travel bans were imposed; no Malaysians were allowed to travel overseas, and tourists and foreigners were not permitted to enter the country. The Malaysians returning from overseas were subjected to health screening and a mandatory 14-day quarantine. Stringent enforcement was implemented, and those who were found violating the MCO rules were subjected to a fixed RM 1000 (approximately USD 247) fine or imprisonment for a maximum of six months as stipulated under Act 342 [15,16,17,18].

The implementation of the MCO was reviewed regularly, and adjustments were made in phases. As of 31 December 2020, Malaysia had experienced four phases of the original MCO (18 March to 12 May 2020), followed by the less stringent Conditional Movement Control Order (CMCO) (13 May to 9 June 2020) and the Recovery MCO (RMCO) (10 June to 31 December 2020) [18]. Despite a relaxation in control measures, stricter MCO rules could be enforced again if a resurgence in positive cases were to occur.

The MCO measures effectively reduced the number of active COVID-19 cases compared with the pre-MCO period [19]. Nevertheless, the unprecedented lockdown substantially affected the daily lives and social relations between citizens. Along with the fear of infection, the lockdown possibly had adverse impacts on mental health [20,21]. Social media platforms were widely used to keep people informed about the pandemic but at the same time accelerated the spread of misinformation, causing public fear and uncertainty [22]. Additionally, perceptions of the government and public responses to the pandemic were directly associated with the mental well-being of the people [23]. Hence, it is vital to study the public’s views and attitudes as well as their compliance with the MCO regulations. This study aimed to understand the public’s perception and experiences of the MCO during the early phase of the COVID-19 pandemic in Malaysia. The findings may provide valuable input to policymakers, which they can use to create effective crisis preparedness and prevention plans.

## 2. Materials and Methods

### 2.1. Study Design 

This exploratory qualitative study consisted of in-depth interviews (IDIs) with 23 Malaysian adults from different demographic backgrounds. The interviews aimed to elicit the participants’ diverse experiences during the early stage of the COVID-19 pandemic when the MCO was implemented in Malaysia. 

### 2.2. Study Setting 

Malaysia enforced the MCO nationwide to curb the spread of COVID-19 in the community. During this period, the Malaysians were confined to their homes and only allowed to travel within a 10 km radius from home for essential or emergency reasons. The participants included in the study were selected purposively to capture their various experiences and encounters in different situations in which they needed to make choices during the MCO implementation. The potential participants were identified from the existing contacts of the research team; however, the interviews were conducted by team members who did not know the participants. The included participants shared some key characteristics: they were Malaysians over 18 years of age and (1) did not have any known close contacts with COVID-19 patients, (2) tested negative for COVID-19, (3) had close contact with those who tested negative for COVID-19, (4) organized mass gatherings during the outbreak of COVID-19, or (5) cancelled events because of the COVID-19 outbreak. Individuals who tested positive for COVID-19 were excluded from the study. 

### 2.3. Data Collection

The data were collected between March 2020 and July 2020. Overall, 23 IDIs were conducted. As the nationwide MCO enforcement had been in effect since 18 March 2020, face-to-face interviews were limited by restricted physical accessibility. Phone interviews were thus employed for all IDIs in this study, except for one face-to-face IDI that was conducted at the workplace as requested by the participant, adhering to physical distancing recommendations. Informed consent and permission to record the audio of the interview were obtained before the IDIs were conducted in person or over the phone. An interview guide containing semi-structured questions was used to facilitate the IDIs, with questions focusing on the “understanding of COVID-19” and “perceptions about public health interventions to control the spread of COVID-19”, followed by probing questions such as “their roles during the COVID-19 pandemic and the MCO”. Information saturation was achieved when no new information could be added to the participant’s perception of the MCO.

The interviews were held at the convenience of the participants. The interviews were conducted either in English, Malay, or Mandarin or a mixture of these languages (as is the norm in Malaysia) depending on the participant’s preference. The sessions lasted between 25 and 110 min. Interview sessions were conducted by research team members who were trained in qualitative methods. The interviewers recorded and took field notes during the IDIs. No repeat interviews were conducted since information gaps were probed in subsequent interviews. All the IDIs were transcribed verbatim and crosschecked against the audio recording by another team member to ensure accuracy of the transcripts. Confidentiality was ensured by removing participants’ identifiers from the transcripts. 

### 2.4. Data Analysis

The data were immediately transcribed verbatim using Microsoft Word and analyzed after each interview. All the transcripts were subsequently uploaded to NVivo 12™ (QSR International, Doncaster, Australia) to facilitate data management and the coding process. The interviews conducted in Malay were transcribed without translations and coded in English, whereas the interviews conducted in Mandarin were translated to English for analysis by a researcher who was a native speaker of Mandarin and subsequently verified by the interviewers. For report writing and dissemination, selected Malay quotes were translated into English. The transcripts were first coded using an open coding method before subsequent grouping during second-order coding. The thematic analysis approach was used to determine the themes and subthemes, and the results were supported by quotations from the participants.

The coding process was conducted independently in pairs, with the same transcript read and coded separately by two researchers, followed by consensual validation between the researchers before regrouping the codes and verbatim quotes into major themes. Throughout the data analysis process, the codebook was continuously revised, with additional subdomains and their definitions added or removed.

## 3. Results

A total of 23 in-depth interviews were carried out among individuals of different demographic backgrounds as shown in Table 1, comprising 16 women and seven men over 21 years of age. Of the 23 participants, 39% worked for the government, 39% worked in the private sector, and 22% were retirees.

Three broad themes emerged from the analysis: a period of information surge, heterogenous emotional response, and attempts to adapt. Several subthemes were identified within each major theme. The themes and subthemes are summarized in Table 2. 

### 3.1. A Period of Information Surge

The participants learned about the MCO from the television, newspapers, and social media. Their responses highlighted the importance of effective communication during a crisis. When public safety and health are concerns, the public needs to know what actions to take to protect themselves. Instructing information in the form of announcements or guidelines helped the public safeguard themselves against a crisis; hence, this information must be clear, efficiently delivered, and accurate. However, information explosion may give rise to confusion among the public.

#### 3.1.1. Source of Information

The government used various platforms, including social media, to reach the public and convey messages or information regarding the disease, preventive measures, and new processes or procedures enacted during the implementation of the MCO. The participants mentioned that they received updated information about COVID-19 and the MCO through several communication platforms. One of the participants stated, “*I think these SMSs (messages sent via SMS by the National Security Council) are a good idea, but maybe the deployment effectiveness could be tweaked a little bit so that people are actually reading (the SMS)…But then there is Telegram…the Telegram from the Ministry of Health is quite good…*” (P14, male, 33 years old).

These platforms also allowed the Malaysians overseas to seek information regarding the procedures for entering the country; thus, they could make an informed decision prior to returning to Malaysia. “*If we want to get the latest news from Malaysia, (we can) follow…(the) Department of Information’s Telegram…for all the latest info…*” (P23, female, 61 years old).

#### 3.1.2. Confusion about Information

Proliferation of misinformation: The proliferation of misinformation can create panic among the public. When the MCO was first announced, unclear information about food stock availability caused rumors to circulate and subsequently triggered panic-buying behavior. One participant said, “*…everybody started saying that shops are going to be closed, so you better go now and buy the things and keep the stock…*” (P22, female, 61 years old).

Unclear information: Clear communication was essential to confer understanding of the rules with which people need to comply. The participants mentioned that unclear information and frequent changes of the MCO rules may have been confusing. One participant stated, “*I think the problem with our (the government’s) announcement is that nobody really knew what to do (or) how to do (it)…*” (P14, male, 33 years old).

Repetition of information: Information disseminated once or twice may not necessarily reach its targeted audience. The participants expressed the need for repetition of the government’s communications during the MCO, as key messages might be lost or go unheard. Repetition of messages could reduce the loss of information when transferred from person to person and decrease misinformation. A participant mentioned, “(*Three or four weeks) after the MCO was announced, people still panicked, and (there was) no proper guidance…some people were saying this, some people were saying that. Even now, for example, with the Gerak Malaysia (a mobile application), the new app, whoever (is) stuck in the kampung (hometown) can travel. Still, people (are) not aware, so I need the government to repeat, repeat, repeat—only then can Malaysians understand…*” (P15, male, 35 years old).

### 3.2. Heterogeneous Emotional Responses

#### 3.2.1. Sense of Seriousness

The participants acknowledged the need for the government to impose the MCO, which was able to break the chain of COVID-19 transmission in the community. They pointed out that the COVID-19 virus was transmitted from person to person; thus, confining people to their homes through the MCO would minimize people’s movement and reduce the spread of the virus. One participant expressed the feeling of safety with the implementation of the MCO measures, “*I really think they (the government) should implement lockdown because it restricts movements. At least we know that when there are cases, the government will lock down that place. People at a place with cases cannot come out. So, I think that would be safer…*” (P21, female, 45 years old).

The participants recognized that compliance with the MCO rules would safeguard high-risk groups, such as the elderly, infants, and low-immunity groups, from contracting the virus. Thus, they acknowledged that it was important to protect these groups from the disease by restricting any form of social visits or unnecessary travel. One participant described her experience as follows: “*Before this outbreak, I usually went out on my own, wherever I wanted to go—I went by bus, I took MRT, and (I) usually went shopping…now, everything stops, I have to stay at home, my niece doesn’t let me go out, my age is 50 and above…(there is a) high risk for me, so she never lets me go out. She will buy (items) for me if there is anything I want…*” (P22, female, 61 years old).

#### 3.2.2. A Need to Be Responsible

The MCO caused major changes in the daily routines of the participants; however, they believed that it was their duty as citizens to abide by the government’s recommendations and fully cooperate in the effort to curb the disease. Due to the physical distancing policy during the MCO, most shops limited the number of shoppers on the premises; thus, queues were often observed outside stores. Nevertheless, the participants indicated the need to comply with the rules despite all the inconveniences: “*It looks like we are wasting time, but we must understand, from the responsibility side, why are they doing this. So, we must tolerate, we must follow; if we need to queue, we must queue because it’s not only me who want to buy things. Many people behind me also are queuing—I cannot enter (the shop) first. Everyone has to follow the rules…*” (P12, female, 34 years old).

#### 3.2.3. Stress

During the MCO period, the participants indicated that the expectation to be in front of the computer and available for online meetings when working from home may lead to stress if this expectation is not monitored properly. One participant said, “*…it was stressful after a long time because when working from home, people expect us to work—we are always online, always sitting in front of the PC (personal computer)*” (P19, male, 30 years old).

#### 3.2.4. Loneliness

As all the Malaysians apart from frontline workers (such as healthcare personnel) were urged to stay at home during the MCO period, the participants mentioned that their physical activity of social interactions decreased, and they felt socially isolated in terms of in-person interactions. “*(We) can’t meet relatives and (we) can’t go out for walks like before. We are not able to travel…*” (P21, female, 45 years old).

The feelings of loneliness, stress, and anxiety due to social isolation were experienced by some of the participants. They expressed being anxious when separated from their loved ones during the MCO because of work-related arrangements. The participants also stated that although they could find new activities to fill the time, being physically alone could lead to loneliness and depression. One participant stated, “*People who live alone have been isolated for two weeks or even more, so it (the MCO) can drive everybody ‘cuckoo’ (crazy)…people can go crazy… you’ll need to address that as well…lots of depression…lots of people getting depressed…*” (P3, female, 64 years old).

#### 3.2.5. Fear of Uncertainty

The participants expressed the fear of the unknown, which could affect their health and the economy. One participant said, “*It (COVID-19) may hit us, and we may get hurt from it. For example, my training (human resource development training) is affected and frozen momentarily. So, many people have lost their jobs, some have permanently lost (their jobs)…*” (P16, female, 58 years old). 

#### 3.2.6. Frustration

The participants observed instances of noncompliance with the standard operating procedure (SOP) of the MCO among other people. These individuals were seen involved in public gatherings or leaving home for non-essential activities. Hence, the participants argued that the implementation of the MCO could be strengthened by the involvement of strict law enforcement to deter the public from violating the SOP: “*I think, in Malaysia, as we heard, people don’t really bother with this epidemic, and they continue their lifestyle as if nothing happened. I think the way the government handles this by using enforcement is a good idea to cater to this group*” (P5, male, 29 years old).

### 3.3. Attempts to Adapt

#### 3.3.1. Alteration of the Working Environment

The MCO caused changes in the routine working life, which was reflected by several participants who had to work remotely from home during the MCO. One participant mentioned, “*…life was affected by my work; I have to work from home…the new (working) style affected me…*” (P20, female, 59 years old).

#### 3.3.2. Utilization of Digital Technologies

The participants experienced separation from their loved ones during the MCO. However, the use of social media platforms, such as Facebook, video calls, and other digital platforms, helped connect them. One participant claimed, “*we are very fortunate to be in the time when we have WhatsApp, a way of staying connected…today, technology is something I’m very thankful for…*” (P16, female, 58 years old).

The utilization of digital technologies was also seen in routine household-related activities. The participants mentioned that they switched from physical grocery shopping to online platforms following the movement restrictions during the MCO. One participant mentioned, “*…that’s why I buy everything online; I changed from my trend of going out to buy things—from going out to buy groceries once a week to buying things online…*” (P18, female, 33 years old).

#### 3.3.3. Opportunity for New Activities

Some of the participants shared their experiences of reorganizing their time by trying new activities during the MCO period. Some of them ventured into cooking, gardening, and meditating to reduce the feeling of boredom and isolation when staying at home during the MCO: “*The benefit (of the MCO) is…to stay at home because (we) can test various recipes. (We) can take care of ourselves and know about ourselves and our health condition…*” (P12, female, 34 years old).

## 4. Discussion

This study was conducted during the early phase of the national Movement Control Order in Malaysia, providing insight into the public’s perceptions and experiences of the government’s response towards the COVID-19 pandemic. This could potentially help the government make better-informed decisions about interventions that are more acceptable to the public. 

Our study suggested that the participants acknowledged the importance of the MCO in breaking the chain of transmission and safeguarding high-risk groups. The participants obtained updates about the disease and the public health interventions primarily through mass and social media, and they preferred information from the government, especially sources from the National Security Council (NSC) and the National Crisis Preparedness and Response Centre (CPRC) under the Ministry of Health (MOH). This was in line with another study conducted in Malaysia, which indicated that those who relied on information from the local authorities, such as the MOH and NSC, had higher confidence and a more positive perception of public health intervention strategies [24]. 

Nevertheless, our participants suggested the need for clear instructions and information dissemination about the MCO, as well as repetition of instructions about the MCO by the government. The success of public health measures, such as the physical distancing recommendations and lockdown policy, depends greatly on the compliance of the public, which in turn relies on one’s awareness and perceived risk of the disease [25]. During a disease outbreak, an effective communication strategy is crucial in shaping the public’s perception of risk and subsequent health behaviors; such a strategy involves clear messages that are distributed across appropriate channels, targeted at varied audiences, and shared by trusted individuals [26,27]. 

Clear and accurate information allows individuals to make choices and take actions to protect themselves, their families, and their communities from threatening health hazards [28]. A trusted source of information is pivotal for the public to understand the disease and comply with preventive measures related to COVID-19. Besides traditional media, such as radio, television, and newspapers, individuals received information through news circulated on the Internet and social media platforms [29]. The media play a key role in public health communications through disease updates, timely dissemination of intervention information by the authorities, and promotion of health and hygiene practices [30]. 

Apart from clear and complete information, evidence from previous studies demonstrates that unambiguous health instructions and effective communication are important in maintaining public trust in response to COVID-19 [31,32]. Trust is a vital component of managing a threat because it affects the public’s judgement about the risk and the related benefits [33]. A previous cross-national study suggested that risk perceptions of COVID-19 were lower among individuals who had a higher level of trust in the government [34]. Additionally, a higher level of trust in the government has been linked to higher compliance with preventive measures [31,35]. As it is critical for the public to trust the government, the government should be well-organized in battling COVID-19 and communicate effectively during crises.

Although rapid circulation of information through social media allows the public to be informed about the government’s interventions and act accordingly, it may also increase the diffusion of misinformation, thus creating panic and fear among the public [32]. In addition, misinformation can distort people’s risk perception about the disease and potentially lead to the rejection of information from the authorities [36,37]. In an effort to mitigate misinformation, several studies proposed guidelines for information dissemination through social media and the establishment of working groups responsible for combating myths and misinformation in various communication platforms [38,39]. In Malaysia, the government provided a “fake news” debunking platform, to which the public could submit any stories circulated on social media that required clarification; the post or article in question would then be verified by relevant authorities and clarified on the website “sebenarnya.my” (translated as “the truth”) [40].

In order to curb the pandemic, our participants felt that it was their responsibility as citizens to comply with the MCO regulations. This might be attributed to the Asian culture of the Malaysian society, which is mainly collectivistic in nature. Contrary to the individualistic cultures, people from a collectivist cultural background tend to view themselves as members of a group and value group goals and social harmony. They care about social recognition and are thus influenced by normative pressures, driving higher levels of compliance [41]. 

Notably, the participants expressed their frustration with those violating the SOP during the MCO, and they suggested the need for stricter enforcement by the authorities. A previous quantitative study conducted in Malaysia indicated that most respondents complied with the MCO because they believed it was their civic duty and because of the fear of being caught and punished by the authorities [42]. Strict mobility restrictions have been shown to be highly effective in combating the spread of COVID-19 in countries such as China and New Zealand [43,44]. Although stringent enforcement of the MCO may be able to ensure compliance among the people, extensive awareness campaigns on the importance of adhering to the MCO should also be promoted by the government. Self-internalization, in which individuals comply with public health interventions voluntarily rather than through external rewards or punishment, can increase people’s adherence to the rules and, ultimately, break the chain of transmission in the community [42,45]. 

The implementation of the MCO caused changes in people’s daily lives. Our study highlighted that alterations in the individuals’ working environments, reduced social (in-person) interactions, and uncertainty about their health and the economy during the MCO had a negative psychological impact on the participants. These findings are similar to those reported in the United Kingdom and Nepal, where physical distancing and isolation policies during the COVID-19 pandemic had adverse effects on the public’s psychological and emotional state because of the lack of in-person social interactions, loss of income, and lifestyle changes [5,46]. Another study in Malaysia reported that two-thirds of respondents suffered from moderate-to-severe anxiety during the early phase of the pandemic because of the perception of high severity and susceptibility to COVID-19 [47]. Furthermore, lockdown measures limiting in-person social interactions could lead to loneliness, thus increasing the prevalence of emotional disturbance, self-harm, and suicide attempts [48,49]. Hence, the government and health authorities should investigate ways to formulate mitigation measures to manage these negative psychological impacts in case re-implementation of movement restriction interventions is required in the future. This will necessitate an increase in the capacity of mental health and psychological support services, an effective communication strategy for public health policies or guidelines to alleviate negative feelings within the community, and the introduction of a job retention scheme to help those who lost their jobs during the MCO period [50,51,52]. 

Moreover, our participants shared their stressful experiences with the unclear boundaries between work and personal life, and they explained that they were expected to always be available when working from home. As working from home will likely become the “new normal” during the pandemic, there is a need to incorporate formalized work-from-home policies for both employers and employees; these policies must address work–home boundary management support, role expectations, responsibilities, technical support, coworker networking facilitation, and other relevant guidelines [53,54,55].

Despite the negative psychological consequences, our participants also shared their experiences in adapting to the changes in their work and personal life resulting from the MCO. One adaptation was the use of digital technologies as tools for communication and grocery shopping. The pandemic triggered a worldwide increase in the use of digital technologies for communication and virtual meetings, especially when lockdowns were enforced. This in turn reduced negative feelings, such as loneliness and anger, through social support [56,57]. Furthermore, the use of digital tools and platforms also increased in teleworking, education, e-commerce, and telehealth during the COVID-19 crisis [58,59,60,61]. With the help of digital technologies, many countries developed contact-tracing applications to facilitate the management of COVID-19 cases [62,63,64]. Similarly, the Malaysian government introduced its own contact tracing application, known as “MySejahtera”, which also allowed people to perform health risk assessments for themselves and their dependents, obtain guidance on appropriate actions according to their COVID-19 risk category, and receive up-to-date information and guidelines about COVID-19 [65]. Given that greater digitalization is expected to continue after the pandemic, policymakers must take the necessary steps towards improving the digital service delivery, especially in the digital transformation of healthcare services.

To our knowledge, this is the first qualitative study exploring the public’s views of the MCO during the COVID-19 pandemic in Malaysia. This study captured the perceptions and experiences of the participants with varying demographic characteristics, such as job status and living environment (e.g., urban vs. rural, living alone or with family members), along with the Malaysian citizens overseas who were trying to return home during the pandemic. The study emphasizes the need for an effective communication strategy and psychological support system during a crisis. However, several limitations need to be addressed. First, the scope of the study was limited to adults in the general population and did not take into account the views of students and specific groups. Moreover, we did not interview individuals who failed to comply with the MCO regulations, and thus we were not able to analyze their understanding of COVID-19 and the MCO. Furthermore, our study focused on the perceptions of the public during the early stage of the pandemic in Malaysia; therefore, the potential long-term impact of the lockdown measures could not be addressed. A prolonged unresolved pandemic may lead to pandemic fatigue, causing public demotivation to comply with the recommended preventive measures [66]. This may pose a greater challenge to the government’s effort in battling the pandemic. Hence, understanding the long-term experiences of the public and identifying the challenges and drivers affecting their desire to practice preventive measures is needed to allow policymakers to design effective prevention strategies that are in line with the needs of the public. In addition, the evidence generated in this study is useful to design quantitative research to measure the impact of lockdown interventions on the population at a wider scale.

## 5. Conclusions

This paper explored how the public perceived the first national MCO in Malaysia and their experiences during that period. Our findings suggest that the participants were aware of the needs and benefits of the MCO in curbing COVID-19 transmission in the country. However, people’s understanding of the public health intervention could be enhanced through more effective communication and information dissemination, as well as actions to combat fake news on various communication platforms. People’s mental health was also affected by MCO implementation; therefore, a psychological support system is needed in times of crisis. Understanding people’s views and attitudes towards the MCO intervention may help policymakers design appropriate public health strategies in the future. 

## Figures and Tables

**Table 1:** Demographic characteristics of the study participants (*n* = 23).

Demographics	*n*	%
Gender		
Male	7	30
Female	16	70
Age group (years)		
21–30	3	13
31–40	7	30
41–50	2	9
51–60	5	22
>60	6	26
Occupation		
Civil servant	9	39
Private sector worker	9	39
Retiree	5	22

**Table 2:** The participants’ perception of the implementation of the MCO.

Themes	Subthemes
A period of information surge	Source of information
	Confusion about information
	Proliferation of misinformation Unclear information Repetition of information
Heterogeneous emotional response	Sense of seriousness A need to be responsible Stress Loneliness Fear of uncertainty Frustration
Attempts to adapt	Alterations of the working environment
	Utilization of digital technologies
	Opportunity for new activities

## Data Availability

To protect the privacy of the respondents, the data set that supports the findings of this article is not publicly available. Request for data can be obtained from the Head of Centre for Biostatistics & Data Repository, National Institutes of Health, Ministry of Health Malaysia on reasonable request and with the permission from the Director General of Health, Malaysia.
